# Genome Sequencing Reveals Widespread Virulence Gene Exchange among Human *Neisseria* Species

**DOI:** 10.1371/journal.pone.0011835

**Published:** 2010-07-28

**Authors:** Pradeep Reddy Marri, Mary Paniscus, Nathan J. Weyand, María A. Rendón, Christine M. Calton, Diana R. Hernández, Dustin L. Higashi, Erica Sodergren, George M. Weinstock, Steven D. Rounsley, Magdalene So

**Affiliations:** 1 The BIO5 Institute, University of Arizona, Tucson, Arizona, United States of America; 2 Graduate Interdisciplinary Program in Genetics, University of Arizona, Tucson, Arizona, United States of America; 3 Department of Immunobiology, University of Arizona, Tucson, Arizona, United States of America; 4 School of Plant Sciences, University of Arizona, Tucson, Arizona, United States of America; 5 Department of Molecular Microbiology and Immunology, Oregon Health and Science University, Portland, Oregon, United States of America; 6 The Genome Center, Washington University School of Medicine, St. Louis, Missouri, United States of America; University of Hyderabad, India

## Abstract

Commensal bacteria comprise a large part of the microbial world, playing important roles in human development, health and disease. However, little is known about the genomic content of commensals or how related they are to their pathogenic counterparts. The genus *Neisseria*, containing both commensal and pathogenic species, provides an excellent opportunity to study these issues. We undertook a comprehensive sequencing and analysis of human commensal and pathogenic *Neisseria* genomes. Commensals have an extensive repertoire of virulence alleles, a large fraction of which has been exchanged among *Neisseria* species. Commensals also have the genetic capacity to donate DNA to, and take up DNA from, other *Neisseria*. Our findings strongly suggest that commensal *Neisseria* serve as reservoirs of virulence alleles, and that they engage extensively in genetic exchange.

## Introduction

The genus *Neisseria* is a large group of β-Proteobacteria that are obligate symbionts of humans and animals. At least eight species of commensal *Neisseria* colonize human mucosal surfaces [1], [2]. These sites are also infected by two pathogenic *Neisseria*: *Neisseria meningitidis*, which causes epidemics of meningitis and septicemia, and *Neisseria gonorrhoeae*, a sexually transmitted bacterium. Because of their importance to global public health, research has focused mainly on the two pathogens, leading to the identification of many virulence factors that are important for infection in humans. Recent reports indicate that commensal *Neisseria* also possess virulence genes [3], [4], [5]. However, these studies focused either on a limited number of virulence genes or on only one commensal genome, that of *Neisseria lactamica*. The total virulence gene content of the *Neisseria* genus is unknown.

The rich diversity of *Neisseria* species at human mucosal surfaces raises the possibility of genetic exchange among these bacteria [6], [7]. Indeed, interspecies exchange of antibiotic resistance genes can occur *in vitro* [8]. These considerations, combined with the limited glimpse of the virulence gene content of commensals, led us to undertake a comprehensive analysis of commensal *Neisseria* genomes and to determine the extent of interspecies genetic exchange.

## Results

We generated high quality draft genome sequences of eight species of human commensal *Neisseria*, and compared them to 11 published *N. meningitidis*, *N. gonorrhoeae* and *N. lactamica* genomes (see Table S1 for all strains used and their corresponding accession numbers). Commensal genomes were sequenced using a combination of the Roche 454 and Illumina platforms. Commensal genomes range between 1.8 and 2.8 Mb and contain 2,000 to 2,842 predicted genes. In contrast, pathogen genomes are ∼2.2 Mb and encode ∼2,000 predicted genes (Table S2). Genes for common metabolic processes, DNA replication, recombination, transcription and translation are conserved in all commensals (Data Set S1 and Text S1).

**Figure 1:**
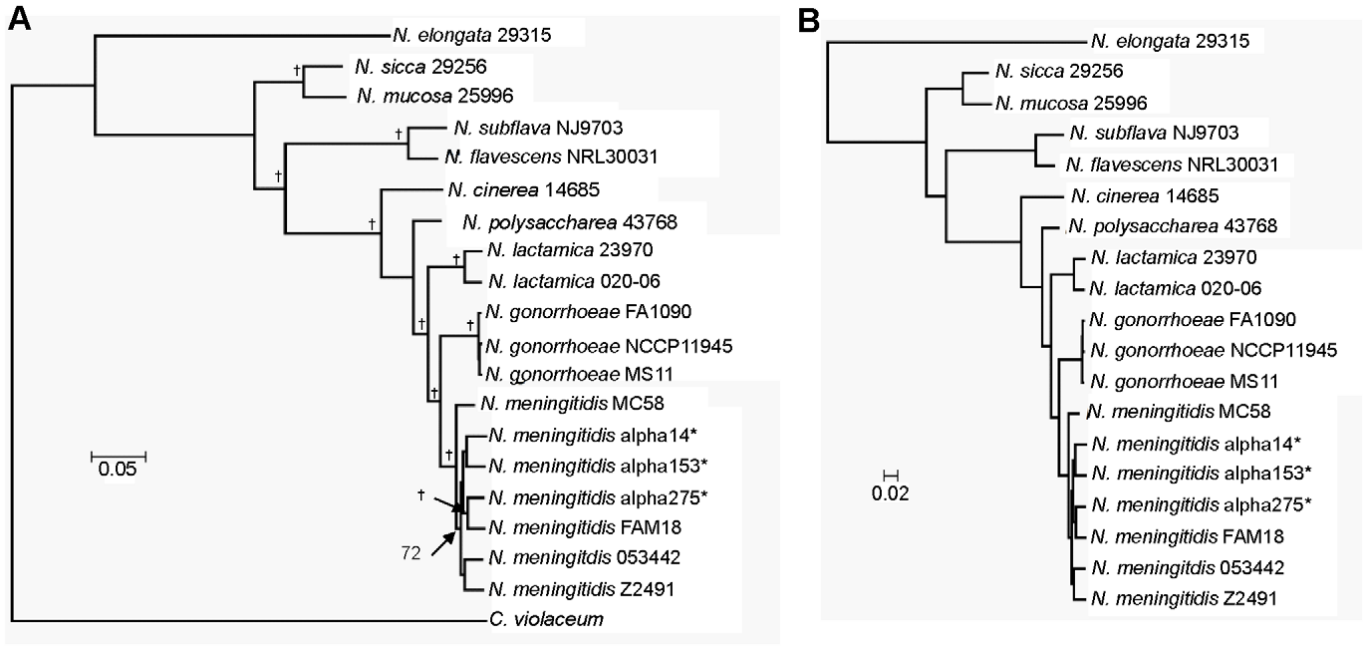
Phylogenetic relationship of human *Neisseria* species. (A) Rooted maximum likelihood *Neisseria* species tree (GTR + I + γ) based on concatenating the DNA sequences of a subset of 636 core *Neisseria* genes that are shared with the outgroup *C. violaceum*. A dagger denotes a bootstrap value of 100. The three *N. meningitidis* carrier strains are denoted by asterisks. (B) Maximum likelihood *Neisseria* species tree (GTR + I + γ) based on concatenating the DNA sequences of all 896 core *Neisseria* genes.

The core *Neisseria* genome – the set of genes present in all *Neisseria* species – consists of 896 genes, mostly with housekeeping function assignments. To determine the phylogenetic relationship of *Neisseria* species, we concatenated the DNA sequences from a subset of 636 *Neisseria* core genes that are shared with *Chromobacterium violaceum*, the outgroup used to root the tree. The *N. meningitidis* and *N. gonorrhoeae* genomes form a distinct monophyletic clade that is derived from the commensal genomes (Figure 1A). The topology of this tree is consistent with that generated by concatenating all 896 *Neisseria* core genes (Figure 1B), and with previously derived single gene phylogenies [2].

**Figure 2:**
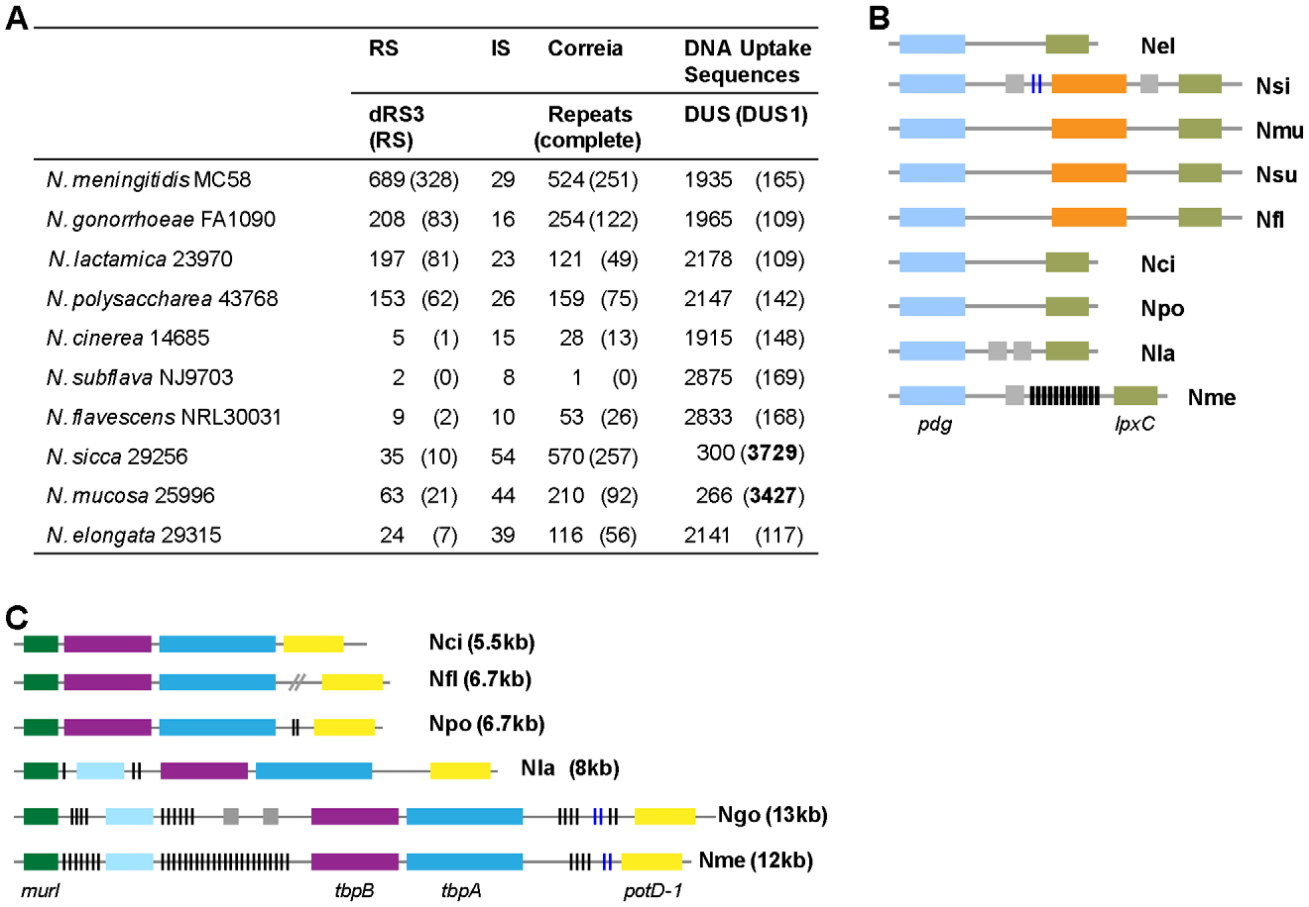
Prevalence of repetitive elements in human *Neisseria* genomes. (A) Numbers of repeat sequences (RS), insertion sequences (IS), Correia elements, and DNA Uptake Sequences (DUS) are given for each *Neisseria* species, with complete copies in parentheses. DUS refers to the canonical DNA uptake sequence GCCGTCTGAA. DUS1 refers to the DUS variant GtCGTCTGAA. Note the high copy number of DUS1 in *N. sicca* and *N. mucosa*, highlighted in bold. (B) Absence of repeat elements in the *pgd/lpxC* locus region in commensals. Nel: *N. elongata*; Nsi: *N. sicca*; Nmu: *N. mucosa*; Nsu*: N. subflava*; Nfl: *N. flavescens*; Nci: *N. cinerea*; Npo*: N. polysaccharea*; Nla: *N. lactamica* 23970; Nme: *N. meningitidis* MC58. Black bars represent dRS3 elements; blue bars represent Correia elements. Orthologous genes are the same color; orange represents a γ-glutamyltranspeptidase present in four commensal genomes; grey represents hypothetical proteins unique to each genome. (C) Absence of large repeat arrays in the *murI-tbpA/B-potD-1* locus in commensals. Ngo: *N. gonorrhoeae* FA1090. Forward slashes (//) represent a break in chromosome contiguity. Orthologous genes are the same color; light blue denotes a hypothetical protein. All other features are the same as in (B).

Repetitive elements are important features of pathogenic *Neisseria* genomes, playing key roles in genetic transformation, gene expression and genome rearrangements [9], [10]. The 10 bp DNA Uptake Sequence (DUS) GCCGTCTGAA is essential for genetic transformation of *N. meningitidis* and *N. gonorrhoeae*. As in pathogenic *Neisseria*, the DUS is the most prominent repetitive element in commensals, occurring over 2,000 times in most species (Figure 2A). Intriguingly, *Neisseria sicca* and *Neisseria mucosa*, which form a distinct clade on the *Neisseria* species tree (Figure 1), have far fewer copies of the canonical DUS. Instead, they have >3,400 copies of a variant DUS (DUS1) with a one base mismatch: GtCGTCTGAA. This DUS1 functions in DNA uptake/transformation in *N. meningitidis* and *N. gonorrhoeae*, albeit less efficiently than the canonical sequence [11], [12]. Thus, all commensals appear to have functional DNA uptake sequences.

Correia and dRS3 elements [13] are the second most abundant repeats in commensal *Neisseria*. These elements function in gene regulation and sequence variation in pathogenic *Neisseria* [14], [15]. Commensals have fewer Correia and dRS3 elements than the pathogens. The only exception is *N. sicca*, which has more Correia elements than either of the pathogens (Figure 2A, 2B and 2C; Text S1). Moreover, commensal dRS3 elements are not arranged into large Intergenic Mosaic Element arrays, unlike their counterparts in pathogenic *Neisseria* (Figure 2B and 2C). This suggests that expansion of dRS3 elements occurred more recently in the pathogens.

In pathogenic *Neisseria*, repetitive elements facilitate two important immune evasion mechanisms: phase and antigenic variation. Many *N. meningitidis* and *N. gonorrhoeae* genes for outermembrane proteins undergo high frequency ON/OFF (phase) switching that results from intragenic recombination of repeat sequences in or near the coding sequence. Over 70% of the genes (52/72) known or hypothesized to be phase variable in pathogenic *Neisseria* [14], [16], [17] are present in commensals (Table S3). However, many of these (26/52) do not have tandem repeats, indicating that recombination-based phase switching of surface proteins occurs less frequently in commensals.

**Figure 3:**
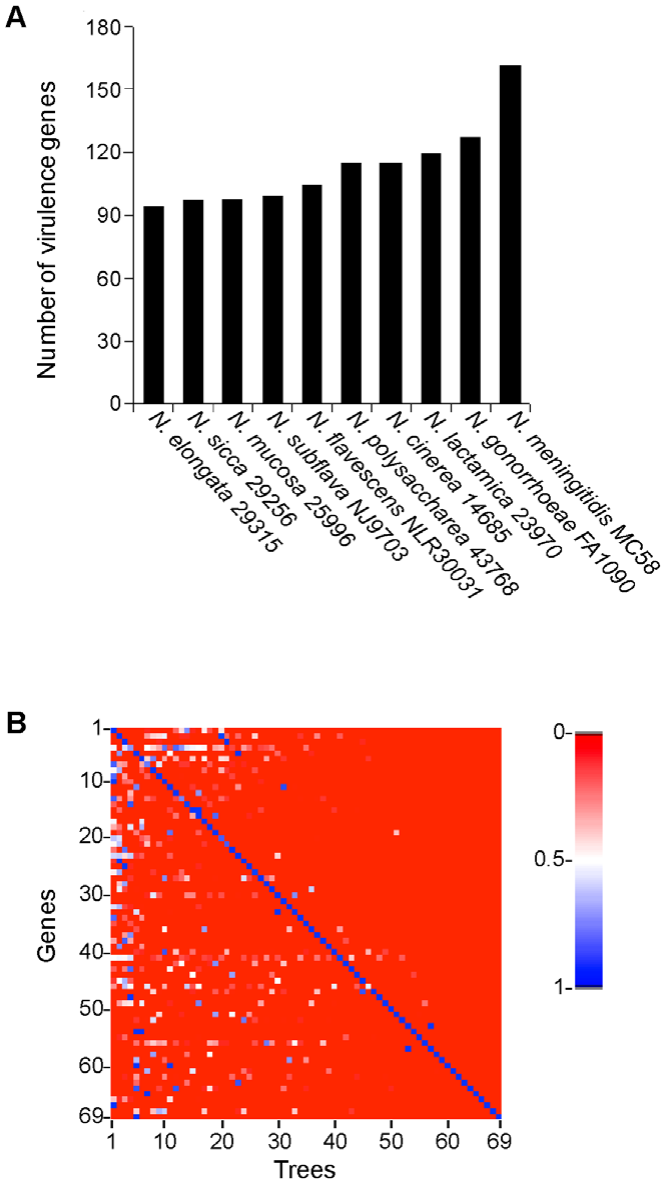
Virulence gene exchange among human *Neisseria* species. (A) Number of genes present in different *Neisseria* species, out of the 177 genes known or hypothesized to be important for virulence (see methods for details). (B) Heatmap of AU test *p*-values obtained by comparing individual virulence gene tree topologies. The map is a 69×69 matrix of 69 virulence genes that are present in all *Neisseria* species (y-axis) and their corresponding topologies (x-axis). Blue indicates a *p*-value of 1 (trees are similar); red indicates a *p*-value of 0 (trees are significantly different).

Studies of a few handpicked genes and whole genome microarrays centered on *N. lactamica* detected virulence genes in commensals [3], [4], [5]. To examine virulence gene content in the newly sequenced commensal genomes, we determined the distribution of 177 genes that have been reported to play a role in *Neisseria* virulence (Table S4) [4], [18], [19]. Seventy-five percent of these genes (133/177) are present in one or more commensals (Figure 3A), and 40% (70/177) are in all of them (Data Set S2). A large subset of *Neisseria* virulence genes is therefore present in the commensals.

Among the virulence genes found in all commensal species are those encoding the Type IV pilus (Tfp), a surface structure that in pathogenic *Neisseria* promotes twitching motility, DNA uptake/genetic transformation, attachment, and host cell signaling [20]. Commensals have a complete set of Tfp biogenesis genes (Figure S1), including: *pilE*, encoding the Tfp structural subunit; *pilD*, encoding a pilin peptidase; *pilF*, encoding a pilin assembly ATPase; and *pilT,* encoding an ATPase required for Tfp retraction and DNA uptake. Commensals also have one or more pilin modification genes. The four commensals closest to the pathogen clade, *N. lactamica*, *Neisseria polysaccharea*, *Neisseria cinerea*, and *Neisseria flavescens*, have a *pilC* ortholog. However, the function of commensal *pilC* cannot be deduced due to sequence differences from pathogenic *Neisseria pilC1* and *pilC2*. Commensals therefore have the genetic capacity to produce Tfp. The functions of commensal Tfp, if expressed, remain to be elucidated.

Tfp systems of commensal and pathogenic *Neisseria* differ in one major respect. In pathogenic *Neisseria*, pilin antigenic variation results from recombination of *pilE* with a silent variant pilin pseudogene, or *pilS* [21]. Pathogenic *Neisseria* have as many as 19 copies of *pilS*, while commensals have only 2–5 copies (Table S5). Moreover, in commensals the region upstream of *pilE* lacks the guanine-repeat element that is essential for *pilE*/*pilS* recombination [22]. Pilin antigenic variation is therefore unlikely to occur in commensals, at least via the guanine-repeat element mechanism. As pseudogenes lacking a function are rapidly lost from the population [23], the presence of *pilS* in commensal genomes is curious. The phylogeny of *Neisseria* (Figure 1) indicates that commensals are basal to the two pathogenic species, which form a single, derived monophyletic clade. The most parsimonious explanation for these observations is that *pilE*/*pilS* antigenic variation is a novel function that arose in the pathogens from a system originally evolved for another, yet unidentified purpose.

The Opa family of outermembrane proteins promote *N. gonorrhoeae* and *N. meningitidis* attachment, invasion, immune cell signaling and inflammation. Pathogen genomes harbor multiple variant *opa*s; *N. meningitidis* has 3–4 *opas*, while *N. gonorrhoeae* has approximately 11 [24]. Most commensals lack *opa* genes and thus do not interact with host cells via this virulence factor. However, *N. polysaccharea*, *N. flavescens* and *N. lactamica* have 1, 2 and 3 variant *opas*, respectively (Table S5). All of these contain variable numbers of the CTCTT pentameric repeat that, in pathogenic *Neisseria*, undergoes slip-strand misrepair resulting in Opa phase and antigenic variation [25], [26]. Commensal Opas are therefore likely to undergo phase switching, but limited antigenic variation.

**Figure 4:**
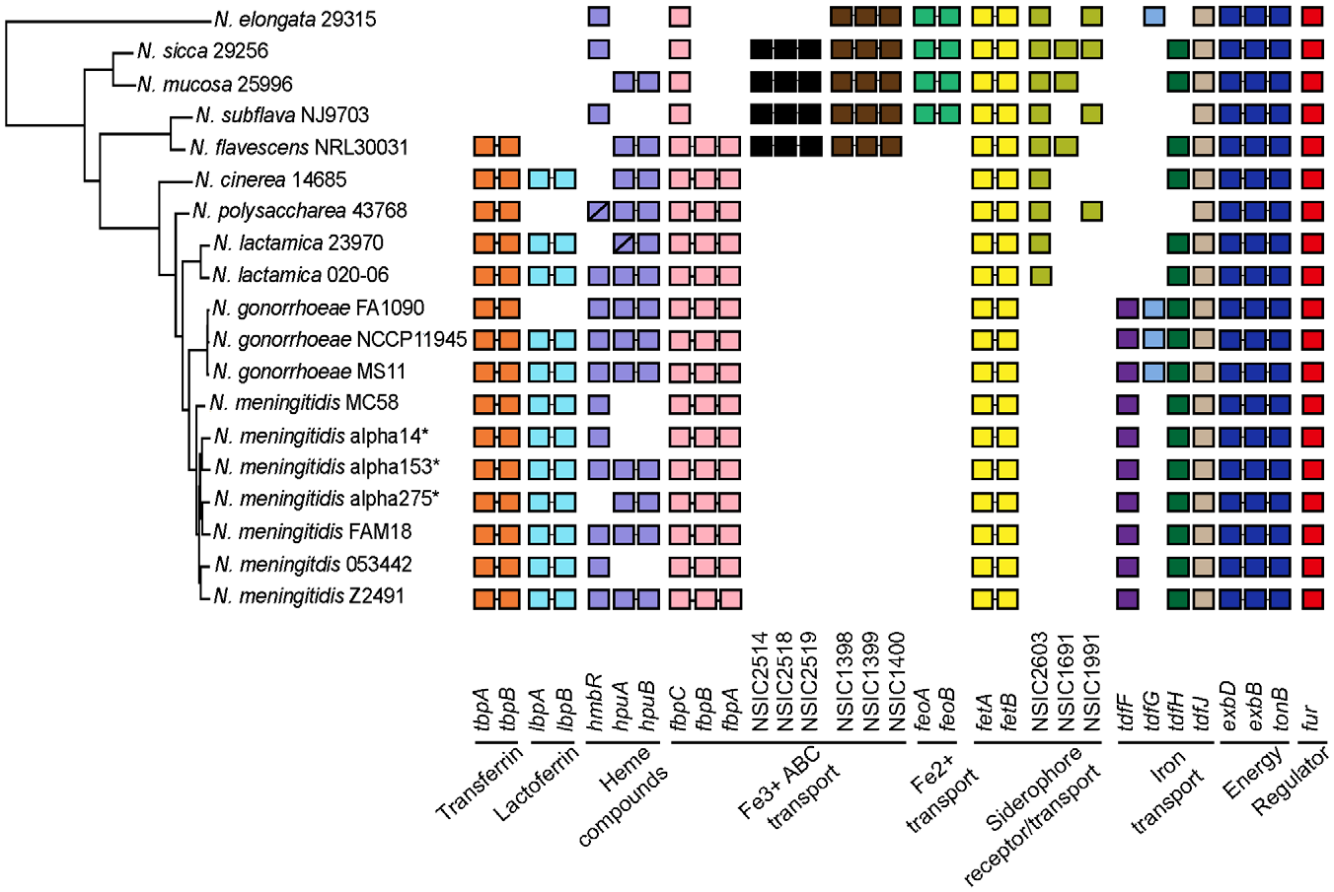
Iron utilization genes in human *Neisseria* species. Species and strain names are listed in the phylogenetic tree. *N. meningitidis* carrier strains are marked with asterisks. Each box represents an individual gene. Genes of the same color belong to the same iron utilization system. Genes connected by a horizontal line are contiguous in the chromosome. Loci confirmed by sequencing to be pseudogenes have a forward slash (/). *N. meningitidis* and *N. gonorrhoeae* gene names appear below each column. Genes are listed as locus tags if their iron utilization functions were deduced from other bacteria. General functions appear below the gene names.

Iron scavenging from the host is an important virulence attribute of bacterial pathogens [27]. Commensal *Neisseria* have a diverse arsenal of iron acquisition genes (Figure 4). *N. lactamica* and *N. cinerea* have genes for acquiring iron from human transferrin and lactoferrin (*tbpA/tbpB* and *lbpA/lbpB*, respectively) [28]. These loci are important for *N. gonorrhoeae* infection and fitness [28]. Commensals also have *hmbR* and *hpuAB*, which are required for acquiring hemoglobin iron [28]. In *N. meningitidis*, HmbR promotes replication in the blood [29]. All commensals have the *tonB/exbB/exbD* iron transport locus, and *fur,* the iron-responsive regulatory element. Notably, commensals most distal to the pathogens have iron uptake genes that are missing from pathogenic *Neisseria*. These include genes for transport of hemin, Fe^+3^ and Fe^+2^, and for siderophore receptors and transport (Figure 4). Because Fe^+2^ is often complexed with protein in foodstuff, the Fe^+2^ uptake system, if expressed, may allow oral commensals to absorb iron from the host diet [28]. Thus, *Neisseria* species possess a diverse array of iron uptake genes. This diversity may help different *Neisseria* species to colonize the same niche without being affected by antibodies directed against the iron acquisition components of other species [30].

Several genes are found uniquely in commensals. Only four genes - encoding hypothetical proteins - are present in all commensals, while several are present in one or more commensals. Intriguingly, some commensal-specific genes encode proteins that function as hemolysins, adhesins or invasins in other bacterial pathogens (Data Sets S3 and S4). Others encode components of the Type IV and Type VI Secretion Systems and lipooligosaccharide modification enzymes (Text S1). Commensal-specific genes are generally smaller, have a lower %G+C content than *Neisseria* core genes, and have homologs in other bacterial genera, indicating that they were acquired recently via horizontal gene transfer.

We also identified several pathogen-specific genes (Data Set S5). Only 16 genes are present in all sequenced *N. meningitidis* genomes, and 81 in all sequenced *N. gonorrhoeae* genomes. Genes for biosynthesis of the polysaccharide capsule, which confers resistance to complement killing and phagocytosis, are unique to *N. meningitidis* (Figure S2). The *ctrABCD* and *lipAB* loci, encoding polysaccharide transporters, are found in some commensals (Text S1). However, the *siaABC* locus, which is required for capsule production, appears only in *N. meningitidis*. The *tdfF* locus which is essential for intracellular iron acquisition and thus for *Neisseria* intracellular replication, is found only in pathogen genomes (Figure 4) [31].

Some genes were found in all sequenced *N. meningitidis* and/or *N. gonorrhoeae* genomes, but only rarely in commensal species. For example, *iga*, encoding an IgA protease that cleaves human IgA and lysosomal membrane protein Lamp1 [20], is present only in pathogenic *Neisseria* genomes with one exception: an intact *iga2* allele is found in both *N. lactamica* genomes. The 57 kb Gonococcal Genetic Island (GGI) [32] is largely absent from commensals. However, genes with varying degrees of nucleotide sequence identity to GGI encoded *dsbC, topB,* and *parA* are present in all commensal genomes (Text S1). In *N. gonorrhoeae*, *dsbC* and *parA* play roles in genetic transformation by secreting DNA [32]. Nf, an M13-like filamentous phage, is the only feature that distinguishes hyperinvasive *N. meningitidis* isolates from noninvasive ones [33], [34]. Nf genes are detected in *N. flavescens* and *N. lactamica*. *N. flavescens* has only a subset of Nf genes, while *N. lactamica* contains all the major M13-like life cycle genes.

Finally, it should be noted that NMB1646, encoding a putative hemolysin, was proposed to be specific to pathogenic *Neisseria*. Microarray studies found NMB1646 in *N. meningitidis* and *N. gonorrhoeae*, but absent from *N. lactamica* [4]. In our study, NMB1646 is also missing from *N. lactamica*. However, it was found in all other commensal genomes. Thus, NMB1646 is present in both commensal and pathogenic *Neisseria*.

Our study shows that commensals have a large number of virulence genes, as well as genes for DNA secretion and uptake, and an abundance of DUS. Additionally, previous work has demonstrated gene exchange among *Neisseria* species [8], [35], [36], [37]. Combined, these results suggest that genetic exchange among *Neisseria* may be more widespread than previously thought. We therefore determined the extent of genetic exchange among *Neisseria* species, using a phylogenetic method to analyze 69 virulence genes shared by all 19 *Neisseria* genomes (Data Set S2). This method relies on the phylogenetic congruence of individual gene trees: a gene tree that does not match the species tree indicates the gene in question has been transferred laterally. We generated individual gene trees for each of the 69 virulence genes using PhyML [38] and looked for the rejection of a set of these topologies by the 69 genes using an Approximate Unbiased (AU) test [39]. The *p*-value heatmap (Figure 3B) indicates that most gene trees showed divergent histories (depicted as groups of topologies with dissimilar *p*-value patterns) that are most likely due to horizontal gene transfer. A small group of topologies (topologies 58–69) were rejected by all the genes. Consistent with these findings, 45% (31/69) of the virulence genes rejected the concatenated tree, compared to 34% of the core genes (average of three replicates of 69 randomly selected core genes). These results suggest that there is widespread genetic exchange among *Neisseria* species, with virulence genes being exchanged at a slightly higher frequency than core genes.

The AU test relies on the rejection of a tree by a gene, rather than on its acceptance, and less divergent or saturated alignments may result in false predictions [40]. Moreover, high rates of recombination between *N. meningitidis* strains can also result in conflicting phylogenies. Therefore, we also looked for evidence of horizontal gene transfer using the program RDPv3.18 [41] to detect homologous intragenic recombination in the 69 virulence genes mentioned above. Evidence of recombination was detected in 53 of the 69 genes, and over half of these events (31/53) involved at least one commensal genome (Table S6). The intragenic recombination and AU tests provide strong evidence of genetic exchange among commensal and pathogenic *Neisseria*, suggesting that commensals act as reservoirs of new virulence alleles.

## Discussion

In this comprehensive analysis of human commensal *Neisseria* genomes, we have identified the gene content of commensal *Neisseria* species, generated an extensive phylogenetic tree of this genus, and determined the distribution of virulence genes in its members. Commensal *Neisseria* have an extensive repertoire of virulence alleles from pathogenic *Neisseria* as well as other bacterial genera. Moreover, they have the genetic machinery for exchanging DNA with each other, and nearly half of *Neisseria* virulence genes have undergone intra- and interspecies recombination. High frequency horizontal gene transfer can increase pathogen fitness, accelerate host adaptation, and affect bacterial virulence. The prevalence of numerous *Neisseria* species in the same niches in the human body provides an opportunity for DNA exchange. The repertoire of virulence genes in *Neisseria* populations is therefore likely to be dynamic.

The large virulence gene set in commensal *Neisseria* raises an intriguing question: why are these bacteria generally not pathogenic? Although there are no ready answers to this question, we can offer possible explanations. First, a productive infection is determined by multiple factors, including the immediate environment of the infection site and host immune status. Commensals may not be able to express the entire constellation of virulence genes necessary for initiating infection under these circumstances. In this context, it is interesting to note that *N. lactamica*, which occasionally causes infections, has the largest set of virulence alleles of all the commensals. Secondly, there may be additional factors governing commensalism that have yet to be defined. *N. meningitidis* can cause asymptomatic infections in humans [42]. Yet, there is very little difference between the genomes of carrier and invasive strains [18]. This suggests that the ON/OFF phase switching of genes may be an important pathogenesis determinant. Indeed, many commensal orthologs of phase-variable genes lack the repeat elements that participate in phase variation. Third, a virulence allele in a commensal may differ from its pathogenic counterpart in its ability to cause disease. For example, *N. gonorrhoeae* expressing the *N. lactamica porB* allele is less able to invade cells [43]. Finally, our results add to the growing body of evidence that suggests the definition of virulence factor needs to be reassessed. Virulence factors have been defined as components whose absence attenuates bacterial virulence but not viability [44]. For example, Tfp is considered a virulence factor because it promotes attachment [45]. However, recent studies demonstrate that Tfp activates pro-survival pathways in epithelial cells [46], [47], a situation that benefits both host and microbe. The presence of Tfp biogenesis genes in commensals supports this idea. Thus, some virulence factors may be more appropriately termed “host adaptation” factors, as they allow bacteria to interact with and adapt to the host environment, but do not specifically promote virulence. The distinction between a true virulence factor versus a host adaptation factor will be clarified as the genomes of more commensal bacteria are sequenced and characterized.

### Accession Numbers

Genome sequences generated in this study were deposited in GenBank. See Table S1 for the GenBank accession numbers for all genome sequences discussed in this paper. Updated versions of the eight commensal *Neisseria* genome sequences generated in this study are available at http://www.u.arizona.edu/~pradeepm/data.html.

## Materials and Methods

### Bacterial strains

The following strains were obtained from the American Type Culture Collection (ATCC): *N. lactamica* (ATCC 23970), *N. polysaccharea* (ATCC 43768), *N. cinerea* (ATCC 14685), *N. sicca* (ATCC 29256), *N. mucosa* (ATCC 25996), and *Neisseria elongata* subsp. *glycolytica* (ATCC 29315). *Neisseria subflava* (NJ9703) was provided by J.B. Kaplan [48]. *N. flavescens* (NRL30031 or H210) was a gift from E.L. Aho [49], [50]. All commensal *Neisseria* strains were grown on chocolate agar with 3.6% GC agar base (BD), 1% Isovitalex (BD), and 1% hemoglobin (Oxoid) for 18–24 hours in 5% CO_2_ at 37°C.

### DNA isolation

Chromosomal DNA was isolated as described previously [51]. Briefly, cells from half of a 10 cm diameter petri dish were harvested with a sterile Dacron swab into 0.5 ml of 50 mM Tris-Cl, 20 mM EDTA, 50 mM NaCl (pH 8). SDS (1% final concentration) and RNAse A (Qiagen, 1 mg/ml) were added, and cells were lysed for five minutes. Two extractions with phenol-chloroform-isoamyl alcohol (25∶24∶1) and one with chloroform were used to remove proteins. DNA was precipitated by adding two volumes of isopropanol followed by suspension in TE (10 mM Tris, 1 mM EDTA, pH 8). Ammonium acetate was added to 2.5 M followed by precipitation with two volumes of ethanol. Following 70% ethanol washes and drying, DNA was suspended in water or 10 mM Tris, 0.1 mM EDTA (pH 8).

### Genome sequencing and assembly

454 sequencing was performed using the Titanium platform (Roche) according to the manufacturer's instructions. Paired-end sequencing was completed using the Illumina Genome Analyzer II following protocols specified by the manufacturer. Draft genomes were assembled using the Newbler (Roche) and Velvet [52] assemblers.

### Genome Annotation

Annotation was performed using a combination of RAST [53] and the Washington University Genome Center's gene prediction pipeline. Select loci were manually curated using previously published *N. meningitidis* and *N. gonorrhoeae* genome sequences as references.

### Orthologs and core genome

Orthologous proteins were identified by BLASTP using reference proteins from previously sequenced *N. meningitidis* and *N. gonorrhoeae* genomes where possible. Predicted proteins were designated orthologs of a reference protein if the ratio of reference self-hit bitscore to predicted protein bitscore was greater than 0.4. The orthology of proteins unique to draft genomes was determined in the same manner, except randomly chosen predicted proteins were used as reference proteins. The orthology was confirmed by genome context whenever required. The 896 genes present in all 19 *Neisseria* genome sequences used in this study were designated as the core *Neisseria* genome.

### Generation of *Neisseria* species tree

The subset of 636 core *Neisseria* genes also present in *C. violaceum* was used to find phylogenetic relationships among *Neisseria* species. The concatenated DNA sequences of these 636 genes were aligned using MAFFT [54] and the resulting alignment edited using Gblocks [55] to remove any gaps. A maximum likelihood tree (using the GTR + I + γ model in PAUP) was generated using the concatenated orthologous sequences from *C. violaceum* as the outgroup. Support for each branch was obtained by performing the bootstrap analysis with 100 replicates. Using the same method, an unrooted tree was also obtained with the concatenated sequences from all 896 core *Neisseria* genes.

### Identification of repeat elements

Repetitive elements of commensal *Neisseria* were identified using the ‘fuzznuc’ application of EMBOSS [56]. DNA Uptake Sequences (DUS) were identified in each genome by looking for the pattern GCCGTCTGAA. Non-canonical DUS were found by allowing one mismatch to the canonical DUS pattern. The dRS3 elements were detected in each genome using the pattern ATTCCCNNNNNNNNGGGAAT [13], [14]. Each identified site was then manually checked to see if it had a lone dRS3 repeat or a complete RS element. Relaxing the search to allow one or two mismatches did not detect any additional RS elements. Correia repeats (CR) were identified by searching for the following patterns [15], [57], with three mismatches allowed per pattern: 

TATAG[CT]GGATTAACAAAAATCAGGAC, TATAG[CT]GGATTAAATTTAAACCGGTAC, TATAG[CT]GGATTAACAAAAACCGGTAC, TATAG[CT]GGATTAAATTTAAATCAGGAC.

Each identified repeat was then manually checked to see if it had a lone CR or a complete Correia element.

### Identification of virulence genes

A list of 177 virulence genes was created by searching published literature and available databases for *Neisseria* genes that are known or hypothesized to have virulence functions [4], [18], [19]. The protein sequences of these 177 genes were used as queries in a TBLASTN search against all commensal genome sequences with an E-value cutoff set at 1e^−5^. A gene was identified as present if it had >50% identity to the query protein and an alignment length of ≥75% of the query length.

### Identification of phase variable genes

Searches of the published literature identified 72 *Neisseria* genes known or hypothesized to be phase variable [14], [16], [17]. The protein sequences of these 72 genes were used as queries in a TBLASTN search against all commensal genome sequences with an E-value cutoff set at 1e^−5^. A gene was identified as present if it had >50% identity to the query protein and an alignment length of ≥75% of the query length. To confirm the presence of the phase variable (PV) repeat, the corresponding gene from the commensal genome sequence was extracted and aligned to the appropriate reference gene sequence. The alignments were manually examined for the presence of the PV repeat.

### Gene tree-to-species tree congruence

A phylogenetic method was used to detect the extent of genetic exchange among *Neisseria* species by examining 69 of the 70 virulence genes that are shared across all 19 *Neisseria* genome sequences. The *pilE* gene was not analyzed, as multiple copies of *pilE* in several of the genomes made identifying orthologs difficult. Phylogenies for each of the 69 genes were derived in PhyML [38] using a GTR + I + γ model of evolution with BIONJ starting tree. The site likelihood for each tree was computed using baseml (PAML package) [58]. The AU test [39] was then applied using Consel [59].

### Detection of intragenic recombination

To provide a secondary method for measuring genetic exchange, the extent of intragenic recombination was also examined. Alignments for each of the 69 virulence genes were generated and recombination breakpoints identified using the program RDPv3.18 [41].

### Confirmation of pseudogenes

Genes of interest were amplified from purified genomic DNA by PCR. All PCR reactions were performed in a 50 µl reaction volume using 50 ng of purified DNA as template, 1X Phusion HF master mix (Finnzymes) and 0.1 mM of the gene-specific primers (Table S7). Thermocycler conditions were as follows: 1 cycle at 98°C for 30 sec; 35 cycles at 98°C for 10 sec, 60°C for 30 seconds and 72°C for 15 sec; 1 cycle at 72°C for 10 min. The resulting PCR products were purified using a column-based method (Qiagen). Purified products were then sequenced using appropriate forward and reverse primers (Table S7) by the Sanger method at the University of Arizona Genetics Core (Tucson, AZ).

## Supporting Information

Text S1 Supplemental text and references.
(0.11 MB DOC)

Figure S1 Human *Neisseria* type IV pilus (Tfp) biogenesis and pilin modification genes. Species and strain names are listed in the phylogenetic tree on the left. *N. meningitidis* carrier strains are denoted by asterisks. Each box represents an individual gene. Genes connected by a horizontal line are contiguous on the chromosome. A forward slash (/) indicates genes that have been confirmed as pseudogenes by sequencing. A backwards slash (\) represents hypothetical pseudogenes whose status has not been confirmed by experimentation. Genes in grey are not involved in Tfp biosynthesis or pilin modification. Tfp genes that have not yet been named are listed by their locus tag designations. The designations of commensal *pilC* orthologs are based on genome context and sequence homology to *pilC1* or *pilC2* of pathogenic *Neisseria*.
(0.89 MB TIF)

Figure S2 Capsular polysaccharide genes of human *Neisseria* species. The *Neisseria* capsular locus is made up of five regions: A, B, C, D, and E. Some sequences also contain a duplicate of the D region, denoted as D'. A composite of the multi-species capsular locus is depicted at the bottom of the figure and includes gene names based on published nomenclature. Each box represents an individual gene, except for numbered boxes, which represent genes of the number listed. Genes of the same color are part of the same pathway; grey boxes denote genes whose functions are unknown or unrelated to the capsule. Genes connected by a horizontal line are contiguous on the chromosome. A backwards slash (\) represents hypothetical pseudogenes whose status has not been confirmed by experimentation. Note that in *N. subflava*, the C and E regions as well as gene NLA0370 are in an inverted orientation compared to the other sequences. Also, whether *N. elongata*, *N. sicca*, and *N. mucosa* contain a D or D' region cannot be ascertained from the available data.
(0.74 MB TIF)

Table S1 Genome sequences used in this study.
(0.07 MB PDF)

Table S2 General characteristics of *Neisseria* genomes.
(0.07 MB PDF)

Table S3 Presence or absence of phase variable genes in commensal *Neisseria*. Targets were identified by searching the literature for genes known or hypothesized to be phase variable in pathogenic *Neisseria*.
(0.17 MB PDF)

Table S4 Genes known or hypothesized to be involved in *Neisseria* virulence. The 177 targets were identified by searching the literature for genes previously identified as having a role in *Neisseria* virulence. Genes in bold type are present in all 19 *Neisseria* genomes used in the study.
(0.13 MB PDF)

Table S5 Copy number of *pilE*, *pilS* and *opa* genes in *Neisseria* species. See supplemental references (Text S1) for additional information on *N. meningitidis* MC58 [3], [20] and *N. gonorrhoeae* FA1090 [20], [21].
(0.08 MB PDF)

Table S6 Recombination of virulence genes among *Neisseria* species. The RDPv3.18 program was used to test for recombination in 69 virulence genes that are shared by all *Neisseria* species.
(0.13 MB PDF)

Table S7 Primers used for PCR amplification and sequencing of potential pseudogenes.
(0.07 MB PDF)

Data Set S1 Common metabolic pathways present in commensal *Neisseria*. Genes from the *N. meningitidis* MC58 genome are included as a reference.
(0.18 MB XLS)

Data Set S2 Virulence genes present in all 19 *Neisseria* genomes. Note that the *N. subflava* and *N. cinerea* genome sequences both contain two copies of *pilE*; the second copy is listed directly below the first (see rows 74–75).
(0.06 MB XLS)

Data Set S3 *Neisseria* strain-specific genes. The name on each tab indicates the species included for the given table.
(0.26 MB XLS)

Data Set S4 Commensal *Neisseria*-specific genes. Genes present in two or more commensal genomes but absent from all *N. meningitidis* and *N. gonorrhoeae* genomes. See supplemental reference [19] (Text S1) for abbreviations of the COG categories.
(0.22 MB XLS)

Data Set S5 Pathogenic *Neisseria*-specific genes. Tab “N. meningitides” includes the seven *N. meningitidis* strains analyzed; tab “N. gonorrhoeae” includes the three *N. gonorrhoeae* genomes studied. See supplemental reference [19] (Text S1) for abbreviations of the COG categories.
(0.04 MB XLS)
