# Supplementary material for: Genome Sequencing Reveals Widespread Virulence Gene Exchange among Human Neisseria Species
Source: PLoS One. 2010 Jul 28;5(7):e11835. doi: 10.1371/journal.pone.0011835 (PMC2911385; doi:10.1371/journal.pone.0011835)
Supplement: Text S1 — Supplemental text and references. (0.11 MB DOC) [file pone.0011835.s001.doc]

**Marri et al. Text S1**

***Supplemental Text***

**Common metabolic pathways.** The genetic components for common metabolic pathways are conserved in commensal *Neisseria* (Data Set S1). Genes for aerobic respiration processes, such as the pentose phosphate pathway and citric acid cycle, are found in all sequenced *Neisseria* genomes. Commensals also encode genes for anaerobic pathways, including those for glycolysis and denitrification [1]. Interestingly, *N. mucosa* alone possesses all the genes (NMUC2098-NMUC2110) necessary for biosynthesis of the cofactor molybdopterin. This suggests that *N. mucosa* is the only *Neisseria* species capable of utilizing molybdopterin-containing enzymes [2].

Genes for nucleotide biosynthesis are conserved in commensal *Neisseria*. Additionally, commensals have transfer RNA genes for all 20 amino acids. All commensals also encode the 16S-23S-5S ribosomal RNA (rRNA) operon. Although our assembly resulted in a single contig for the rRNA genes, the contig depth was four-fold higher compared to the average genome coverage. Thus, similar to pathogenic *Neisseria* [3], the commensals each have at least four copies of the rRNA operon.

In general, commensal *Neisseria* encode the genes necessary for biosynthesis of essential amino acids. However, *N. cinerea, N. polysaccharea*, and *N. flavescens* lack several sulfur metabolism genes that are required for cysteine biosynthesis in other bacteria [4]. This result suggests that these commensal isolates may need to scavenge cysteine from the environment.

**dRS3 and Correia repetitive elements.** The commensal *Neisseria* genome sequences have fewer dRS3 and Correia elements when compared to the pathogens. While the scarcity of these elements may reflect an inability of 454 sequencing to handle repeat-rich sequences, we believe that this explanation is unlikely. The Titanium 454 sequencing method used in this study resulted in average read lengths of >400bp. As most *Neisseria* repeat elements are <250bp [5], the Titanium technology should have been able to handle the repeat lengths found in our samples. To confirm this, we performed two tests. First, we searched the raw 454 reads of each genome sequence to see if we could find additional dRS3 or Correia elements that had failed to assemble. After normalizing for depth of coverage, we did not find any additional repeat elements. Second, we identified genes in the *N. meningitidis* MC58 genome sequence with 10 or more dRS3 elements located between them, and scanned the corresponding orthologous regions in *N. cinerea* for these repeat elements. *N. cinerea* was used because it has the lowest number of contigs of all the commensal sequences. We found that the orthologous intergenic regions in *N. cinerea* lacked dRS3 elements, and that these intergenic regions were smaller on average than the corresponding regions in MC58. These analyses confirm that our findings are not artifacts, and that commensal *Neisseria* have fewer dRS3 and Correia repeats compared to pathogenic *Neisseria*.

**Commensal-specific genes.** Comparison of the sequenced commensal and pathogenic genomes identified several genes that are present in two or more commensals but absent from *N. meningitidis* and *N. gonorrhoeae* (Data Sets S3 and S4). For example, commensals have Type IV and Type VI Secretion Systems not found in pathogenic *Neisseria*, as well as distinct lipooligosaccharide modification genes (see below). Commensals most distal to the pathogens (*N. elongata*, *N. sicca*, *N. mucosa*, *N. subflava*) encode genes for collagenases similar to those found in the oral bacterium *Eikenella corrodens*. These genes have a higher %G+C content than the core *Neisseria* genome (55% vs. 50%) and their predicted products have >50% identity at the protein level to the *E. corrodens* collagenases*.* These observations suggest commensal *Neisseria* may have acquired new proteases from *E. corrodens* via lateral gene transfer.

**Type IV Secretion Systems.** Gram-negative bacteria utilize Type IV Secretion Systems (T4SS) to transport substrates such as DNA and protein effectors across the bacterial cell envelope [6]. The Gonococcal Genetic Island (GGI, see also below) of *N. gonorrhoeae* encodes T4SS genes similar to those on the *Escherichia coli* F plasmid [7]. The GGI T4SS allows *N. gonorrhoeae* to secrete DNA into the extracellular milieu. Two commensal species, *N. mucosa* and *N. sicca*, encode T4SS genes that are most similar to the *Agrobacterium tumefaciens* VirB/D4 system (Data Sets S3 and S4). *N. mucosa* and *N. sicca* have orthologs of the T4SS coupling protein/substrate receptor (*virD4*), ATPases for translocation energetics (*virB4* and *virB11*), components of the channel structure (*virB6*, *virB8*, *virB9*, and *virB10*), and the secreted relaxase that is involved in DNA processing and packaging (*virD2*). As the *A. tumefaciens* VirB/D4 system is involved in DNA transfer, the presence of *vir* genes in *N. mucosa* and *N. sicca* suggests that these commensal *Neisseria* may also utilize a T4SS to export DNA into the environment.

**Type VI Secretion Systems.** Three commensal species, *N. mucosa*, *N. sicca* and *N. subflava*, contain genes encoding Type VI Secretion Systems (T6SS) (Data Sets S3 and S4). Essential for virulence in several pathogens [8,9,10], T6SS are also found in symbionts [11] and commensals [12]. These are the first examples of T6SS in the *Neisseria* genus.

**Lipooligosaccharide modification.** The *lic1* (*licABCD*) locus is present in multiple species of commensal *Neisseria* (Data Sets S3 and S4). In *Haemophilus influenzae* *lic1* encodes genes necessary for phosphorylcholine decoration of lipopolysaccharide [13,14]. Three commensal *Neisseria* species*, N. flavescens*, *N. lactamica*, and *N. subflava*, contain *lic1* and incorporate phosphorylcholine into their lipooligosaccharide (LOS) [15]. We also found *licABCD* in the genome sequences of *N. flavescens* and *N. lactamica*, but not *N. subflava.* The difference in findings from the two studies is most likely due to the use of different strains of *N. subflava*. The *lic1* locus is also present in the genome sequence of *N. polysaccharea*, a species that was not examined by the other group. Thus, *N. polysaccharea* may also modify its LOS with phosphorylcholine.

**Capsule genes in commensal *Neisseria***. Among commensals, the *ctrABCD* locus is present only in *N. subflava*. These genes are inverted relative to the orientation of the meningococcal homologs (Figure S2). A DNA Uptake Sequence (DUS) is present in the *N. subflava* *ctrA* gene, but not in the other *ctrA* homologs. The *N. subflava*DUS differs from the canonical DUS only at one position. Thus, *N. subflava* may have acquired the *ctrABCD* locus through this variant DUS. As DUS is hypothesized to function in genome maintenance, the *N. subflava* variant DUS may be responsible for maintenance of the *ctrABCD* locus in this commensal species.

**Gonococcal Genetic Island (GGI).** The 57 kb GGI is found in most *N. gonorrhoeae* isolates [16]. Several features of the GGI, including a lower %G+C content and fewer gonococcal DNA Uptake Sequences than the rest of the chromosome, suggest that this element was acquired via horizontal gene transfer. The function of many GGI genes is unknown; however, several contribute to virulence by promoting DNA secretion and autolysis [7]. As noted in the main text, three GGI genes, *dsbC, topB,* and *parA,* are found in all sequenced commensal genomes. Other GGI genes are only found in some but not all commensal genomes. For example, *N. flavescens* and *N. lactamica* both contain *yecA* and *yeb*. The former encodes a hypothetical protein, while Yeb is a homolog of a putative *N*-acetyltransferase from *Ralstonia solanacearum* [7]. *N. subflava* contains *ydcB*, which also encodes a hypothetical protein [7]. Finally, a copy of *traD* is found in *N. elongata*. The *traD* gene is encoded on the F plasmid and involved in conjugation; the *Salmonella typhi* TraD is a DNA-dependent ATPase that functions as a DNA pump [17].

It should be noted that GGI genes are also found in one of the *N. meningitidis* genomes we examined. The *N. meningitidis* carriage isolate alpha275 has nearly all the GGI genes, arranged in the same order as those in the *N. gonorrhoeae* GGI. This finding correlates with previous microarray studies that revealed the presence of complete or nearly complete copies of the GGI in several other *N. meningitidis* strains [18].

***Supplemental References***

1. Barth KR, Isabella VM, Clark VL (2009) Biochemical and genomic analysis of the denitrification pathway within the genus *Neisseria*. Microbiology 155: 4093-4103.

2. Schwarz G, Mendel RR, Ribbe MW (2009) Molybdenum cofactors, enzymes and pathways. Nature 460: 839-847.

3. Tettelin H, Saunders NJ, Heidelberg J, Jeffries AC, Nelson KE, et al. (2000) Complete genome sequence of *Neisseria meningitidis* serogroup B strain MC58. Science 287: 1809-1815.

4. Ostrowski J, Wu JY, Rueger DC, Miller BE, Siegel LM, et al. (1989) Characterization of the *cysJIH* regions of *Salmonella typhimurium* and *Escherichia coli* B. DNA sequences of *cysI* and *cysH* and a model for the siroheme-Fe4S4 active center of sulfite reductase hemoprotein based on amino acid homology with spinach nitrite reductase. J Biol Chem 264: 15726-15737.

5. Parkhill J, Achtman M, James KD, Bentley SD, Churcher C, et al. (2000) Complete DNA sequence of a serogroup A strain of *Neisseria meningitidis* Z2491. Nature 404: 502-506.

6. Christie PJ, Atmakuri K, Krishnamoorthy V, Jakubowski S, Cascales E (2005) Biogenesis, architecture, and function of bacterial type IV secretion systems. Annu Rev Microbiol 59: 451-485.

7. Hamilton HL, Dominguez NM, Schwartz KJ, Hackett KT, Dillard JP (2005) *Neisseria gonorrhoeae* secretes chromosomal DNA via a novel type IV secretion system. Mol Microbiol 55: 1704-1721.

8. Mougous JD, Cuff ME, Raunser S, Shen A, Zhou M, et al. (2006) A virulence locus of *Pseudomonas aeruginosa* encodes a protein secretion apparatus. Science 312: 1526-1530.

9. Pukatzki S, Ma AT, Sturtevant D, Krastins B, Sarracino D, et al. (2006) Identification of a conserved bacterial protein secretion system in *Vibrio cholerae* using the *Dictyostelium* host model system. Proc Natl Acad Sci U S A 103: 1528-1533.

10. Schell MA, Ulrich RL, Ribot WJ, Brueggemann EE, Hines HB, et al. (2007) Type VI secretion is a major virulence determinant in *Burkholderia mallei*. Mol Microbiol 64: 1466-1485.

11. Fauvart M, Michiels J (2008) Rhizobial secreted proteins as determinants of host specificity in the rhizobium-legume symbiosis. FEMS Microbiol Lett 285: 1-9.

12. Di Bonaventura MP, DeSalle R, Pop M, Nagarajan N, Figurski DH, et al. (2009) Complete genome sequence of *Aggregatibacter (Haemophilus) aphrophilus* NJ8700. J Bacteriol 191: 4693-4694.

13. Weiser JN, Love JM, Moxon ER (1989) The molecular mechanism of phase variation of *H. influenzae* lipopolysaccharide. Cell 59: 657-665.

14. Weiser JN, Shchepetov M, Chong ST (1997) Decoration of lipopolysaccharide with phosphorylcholine: a phase-variable characteristic of *Haemophilus influenzae*. Infect Immun 65: 943-950.

15. Serino L, Virji M (2000) Phosphorylcholine decoration of lipopolysaccharide differentiates commensal Neisseriae from pathogenic strains: identification of *licA*-type genes in commensal Neisseriae. Mol Microbiol 35: 1550-1559.

16. Dillard JP, Seifert HS (2001) A variable genetic island specific for *Neisseria gonorrhoeae* is involved in providing DNA for natural transformation and is found more often in disseminated infection isolates. Mol Microbiol 41: 263-277.

17. Tato I, Zunzunegui S, de la Cruz F, Cabezon E (2005) TrwB, the coupling protein involved in DNA transport during bacterial conjugation, is a DNA-dependent ATPase. Proc Natl Acad Sci U S A 102: 8156-8161.

18. Snyder LA, Jarvis SA, Saunders NJ (2005) Complete and variant forms of the 'gonococcal genetic island' in *Neisseria meningitidis*. Microbiology 151: 4005-4013.

19. Tatusov RL, Koonin EV, Lipman DJ (1997) A genomic perspective on protein families. Science. 278: 631-637.

20. Dehio C, Gray-Owen SD, Meyer TF (1998) The role of neisserial Opa proteins in interactions with host cells. Trends Microbiol 6: 489-495.

21. Hamrick TS, Dempsey JA, Cohen MS, Cannon JG (2001) Antigenic variation of gonococcal pilin expression in vivo: analysis of the strain FA1090 pilin repertoire and identification of the *pilS* gene copies recombining with *pilE* during experimental human infection. Microbiology 147: 839-849.
