# Supplementary figures and images for: Genome Sequencing Reveals Widespread Virulence Gene Exchange among Human Neisseria Species
Source: PLoS One. 2010 Jul 28;5(7):e11835. doi: 10.1371/journal.pone.0011835 (PMC2911385; doi:10.1371/journal.pone.0011835)

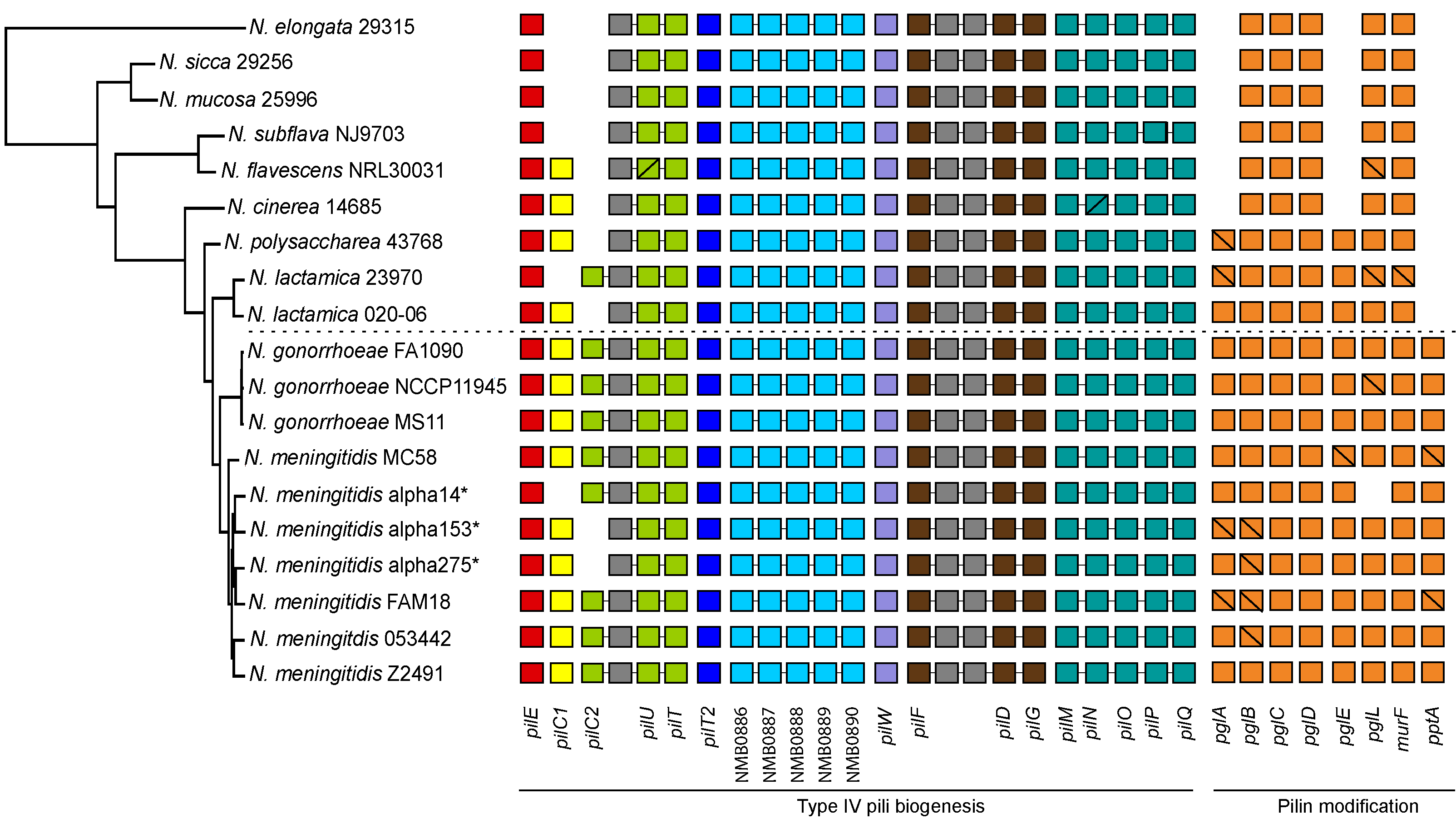

Supplement: Figure S1 — Human Neisseria type IV pilus (Tfp) biogenesis and pilin modification genes. Species and strain names are listed in the phylogenetic tree on the left. N. meningitidis carrier strains are denoted by asterisks. Each box represents an individual gene. Genes connected by a horizontal line are contiguous on the chromosome. A forward slash (/) indicates genes that have been confirmed as pseudogenes by sequencing. A backwards slash (\) represents hypothetical pseudogenes whose status has not been confirmed by experimentation. Genes in grey are not involved in Tfp biosynthesis or pilin modification. Tfp genes that have not yet been named are listed by their locus tag designations. The designations of commensal pilC orthologs are based on genome context and sequence homology to pilC1 or pilC2 of pathogenic Neisseria. (0.89 MB TIF) [file pone.0011835.s002.tif]

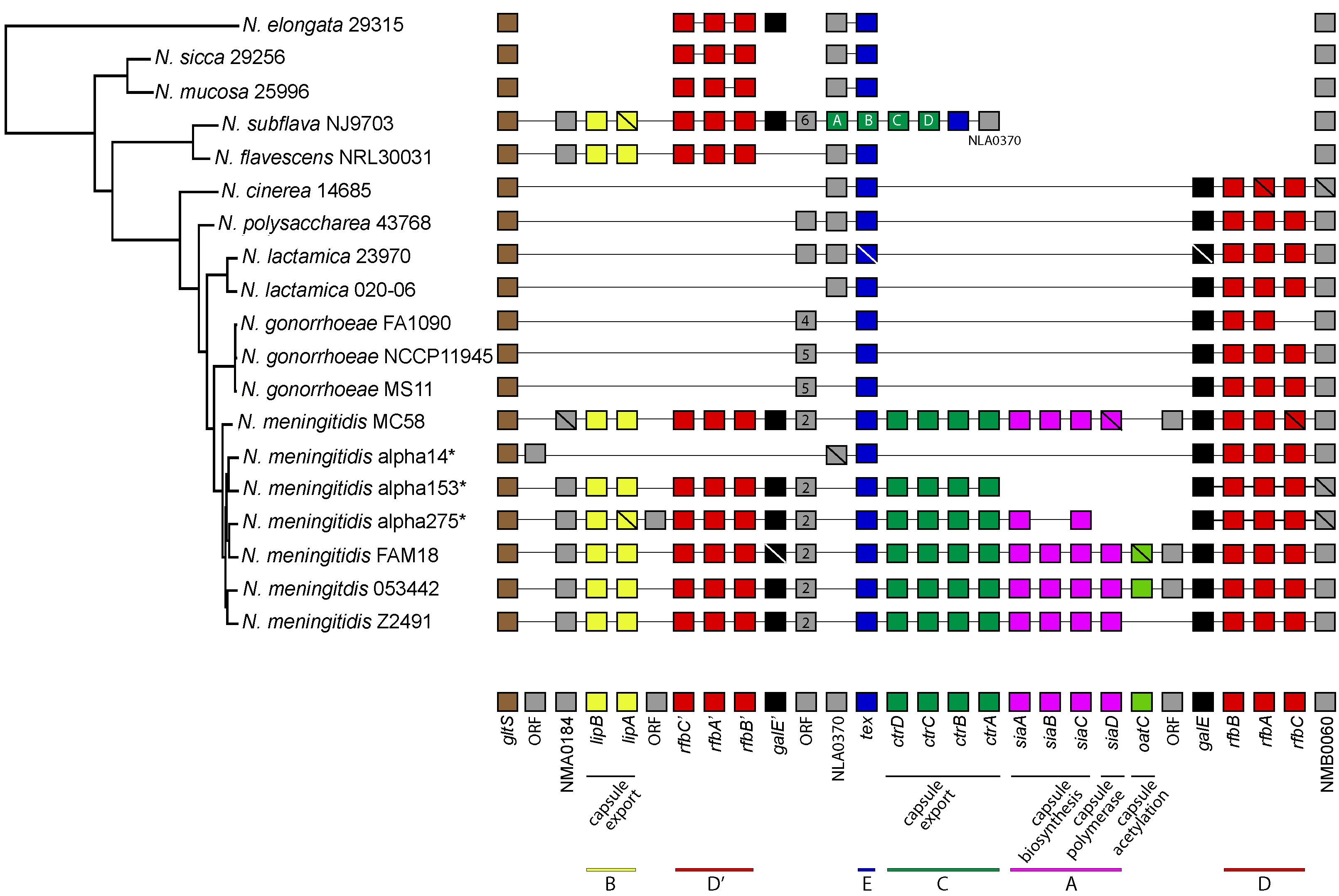

Supplement: Figure S2 — Capsular polysaccharide genes of human Neisseria species. The Neisseria capsular locus is made up of five regions: A, B, C, D, and E. Some sequences also contain a duplicate of the D region, denoted as D'. A composite of the multi-species capsular locus is depicted at the bottom of the figure and includes gene names based on published nomenclature. Each box represents an individual gene, except for numbered boxes, which represent genes of the number listed. Genes of the same color are part of the same pathway; grey boxes denote genes whose functions are unknown or unrelated to the capsule. Genes connected by a horizontal line are contiguous on the chromosome. A backwards slash (\) represents hypothetical pseudogenes whose status has not been confirmed by experimentation. Note that in N. subflava, the C and E regions as well as gene NLA0370 are in an inverted orientation compared to the other sequences. Also, whether N. elongata, N. sicca, and N. mucosa contain a D or D' region cannot be ascertained from the available data. (0.74 MB TIF) [file pone.0011835.s003.tif]
