# Supplementary material for: Genome Sequencing Reveals Widespread Virulence Gene Exchange among Human Neisseria Species
Source: PLoS One. 2010 Jul 28;5(7):e11835. doi: 10.1371/journal.pone.0011835 (PMC2911385; doi:10.1371/journal.pone.0011835)
Supplement: Table S1 — Genome sequences used in this study. (0.07 MB PDF) [file pone.0011835.s004.pdf]

Table S1. Genome sequences used in this study.

#	Species	Strain	Source	Phenotype	Accession #
1	*N. meningitidis*	Z2491	GenBank	Pathogen	NC_003116
2	*N. meningitidis*	MC58	GenBank	Pathogen	NC_003112
3	*N. meningitidis*	FAM18	GenBank	Pathogen	NC_008767
4	*N. meningitidis*	053442	GenBank	Pathogen	NC_010120
5	*N. meningitidis*	alpha14	GenBank	Carriage	NC_013016
6	*N. meningitidis*	alpha153	GenBank	Carriage	AM889137 (draft)
7	*N. meningitidis*	alpha275	GenBank	Carriage	AM889138 (draft)
8	*N. gonorrhoeae*	FA1090	GenBank	Pathogen	NC_002946
9	*N. gonorrhoeae*	NCCP11945	GenBank	Pathogen	NC_011035
10	*N. gonorrhoeae*	MS11	GenBank	Pathogen	NZ_ABZK00000000
11	*N. lactamica*	020-06	Sanger Institute	Commensal	www.sanger.ac.uk/Projects/N_lactamica
12	*N. lactamica*	ATCC 23970	This study	Commensal	NZ_ACEQ00000000
13	*N. polysaccharea*	ATCC 43768	This study	Commensal	ADBE00000000
14	*N. cinerea*	ATCC 14685	This study	Commensal	NZ_ACDY00000000
15	*N. flavescens*	NRL30031 (H210)	This study	Commensal	NZ_ACEN00000000
16	*N. subflava*	NJ9703	This study	Commensal	NZ_ACEO00000000
17	*N. sicca*	ATCC 29256	This study	Commensal	NZ_ACKO00000000
18	*N. mucosa*	ATCC 25996	This study	Commensal	NZ_ACDX00000000
19	*N. elongata* subsp. *glycolytica*	ATCC 29315	This study	Commensal	NZ_ADBF00000000
