# Supplementary material for: Genome Sequencing Reveals Widespread Virulence Gene Exchange among Human Neisseria Species
Source: PLoS One. 2010 Jul 28;5(7):e11835. doi: 10.1371/journal.pone.0011835 (PMC2911385; doi:10.1371/journal.pone.0011835)
Supplement: Table S2 — General characteristics of Neisseria genomes. (0.07 MB PDF) [file pone.0011835.s005.pdf]

Table S2. General characteristics of *Neisseria* genomes.

Genome	Size (bp)	Contig number	Contig N50 (bp)	GC content (%)	Gene number	Avg. gene length (bp)	Gene density (%)
N. elongata	2,260,105	53	97,339	54.2	2589	733.24	84.0
N. sicca	2,786,309	53	122,336	50.9	2842	789.19	80.5
N. mucosa	2,542,952	198	23,441	51.1	2594	802.36	81.8
N. subflava	2,288,219	41	275,495	49.0	2303	848.89	85.4
N. flavescens	2,199,447	80	60,505	49.2	2240	837.48	85.3
N. cinerea	1,876,338	34	179,760	50.8	2050	794.43	86.8
N. polysaccharea	2,043,594	85	106,756	52.0	2268	762.39	84.6
N. lactamica 23970	2,148,211	95	41,107	52.3	2359	778.95	85.5
N. gonorrhoeae FA1090	2,153,922	1	-	52.7	2002	845.35	78.6
N. meningitidis MC58	2,184,406	1	-	51.8	2049	857.13	80.4
