# Supplementary material for: Genome Sequencing Reveals Widespread Virulence Gene Exchange among Human Neisseria Species
Source: PLoS One. 2010 Jul 28;5(7):e11835. doi: 10.1371/journal.pone.0011835 (PMC2911385; doi:10.1371/journal.pone.0011835)
Supplement: Table S3 — Presence or absence of phase variable genes in commensal Neisseria. Targets were identified by searching the literature for genes known or hypothesized to be phase variable in pathogenic Neisseria. (0.17 MB PDF) [file pone.0011835.s006.pdf]

Table S3. Presence or absence of phase variable genes in commensal *Neisseria*. Targets were identified by searching the literature for genes known or hypothesized to be phase variable in pathogenic *Neisseria*.

* Experimentally proven (K) or strong candidate (S) for phase variation.
** Gene used as a query to search for orthologs in commensal genomes. Genes in bold have the phase variable (PV) repeat in the promoter.
*** The nucleotides in the repeat are in parentheses, followed by a number denoting the length of the repeat. A lower case "c" indicates that the repeat is present in the coding region.

aa: gene absent, PV repeat absent
pp: gene present, PV repeat present
pa: gene present, PV repeat absent
p?: gene present, PV repeat has not been defined

Nci: *N. cinerea*
Npo: *N. polysaccharea*
Nla: *N. lactamica* 23970
Nfl: *N. flavescens*

Nsu: *N. subflava*
Nsi: *N. sicca*
Nmu: *N. mucosa*
Nel: *N. elongata*

**Candi-dacy***	**Gene**	**Annotated function**	**Reference gene****	**PV repeat*****	**Gene length (bp)**	***Nci***	***Npo***	***Nla***	***Nfl***	***Nsu***	***Nsi***	***Nmu***	***Nel***
S		Hypothetical protein	**NMA0132**	**(C) 9**	**1056**	**aa**	**p?**	**p?**	**aa**	**aa**	**aa**	**aa**	**aa**
S		Acetyltransferase	NMA0407	(G) 12	569	aa	aa	aa	aa	aa	aa	aa	aa
S		Prolyl oligopeptidase family protein	NMA0529	(T) 5	795	pp	pp	pp	pa	pa	pa	pa	pa
S		Hypothetical protein	NMA0562	(TTCC) 4	882	aa	aa	pp	pa	pa	pa	pa	aa
S		PotD-2	NMA0831	(C) 7	1140	pa	pp	pa	pp	pa	pa	pa	pa
S		IldD	NMA1592	(G) c	1173	pa	pa	pa	pa	pa	pa	pa	pa
S		DnaX	NMA1656	(C) 7	2130	aa	aa	aa	aa	aa	aa	aa	aa
S	*vapA*	Virulence associated protein	NMA2175	(AAGC) 9	1860	aa	pa	pa	pa	aa	aa	aa	aa
**S**		**Putative lipoprotein**	**NMB0032**	**(A) 11**	**528**	**p?**	**p?**	**p?**	**aa**	**aa**	**aa**	**aa**	**aa**
S		Putative hydrolase	NMB0039	(C) 7	273	pa	pa	pa	pa	pa	pa	pa	pa
S			NMB0040	(C) c	714	pa	aa	aa	pa	pa	pa	pa	pa
S		Hypothetical protein	NMB0065	(T) 10	927	aa	aa	aa	aa	aa	aa	aa	aa
K	*siaD*	Capsule biosynthesis protein	NMB0067	(C) 7	678	aa	aa	aa	aa	aa	aa	aa	aa

S		RplK	NMB0127	(C) c	435	pa	pa	pp	pp	pa	pp	pp	pp
K	*pgtA* / *pglA*	Pilin glycosylation protein	NMB0218	(G) 11	1131	aa	pa	pp	aa	aa	aa	aa	aa
K		Hypothetical protein	NMB0297	(G) c	99	aa	aa	pa	aa	aa	aa	aa	aa
S		TspA	NMB0341	(C) 7	2628	pa	pa	pa	aa	aa	aa	aa	aa
S		Hypothetical protein	NMB0368	(A) 11	459	pa	pp	pp	pp	aa	pa	aa	aa
K	*dca*/ *pptA*	Competence-associated protein / pilin phosphorylcholine transferase	NMB0415	(G) 9	1648	aa	aa	aa	aa	aa	aa	aa	aa
S		AmiC	NMB0456		1251	p?	p?	p?	p?	p?	p?	p?	p?
S		Phage associated	NMB0486	(C) 6 (N)10 (G)7	1343	aa	aa	pp	pa	aa	aa	aa	aa
S		Glycosyltransferase	NMB0624	(CAAACAA) 34	1222	aa	aa	aa	aa	aa	aa	aa	aa
S	*hsdS*	Type I restriction enzyme S protein	NMB0831	(G) 7	1174	aa	pp	aa	aa	aa	aa	aa	pa
S		Glycotransferase	NMB0846	(A) 8	1035	pa	aa	aa	pa	pp	pa	pa	pa
S		SodB	NMB0884	(T) 5	588	pa	pp	pp	pa	pa	pa	pa	pa
S		AzlC-related	NMB0892	(A) 6	423	pp	pp	pp	pa	pp	pp	pp	pp
S		FunZ	NMB0961	(CAAT) 5	1596	aa	pa	aa	aa	aa	aa	aa	aa
S		Phage associated	NMB0970	(C) 6 (N)10 (G) 7	1425	aa	aa	pp	pa	aa	aa	aa	aa
K	*opc*	Class 5 outer membrane protein	NMB1053	(C) 12	819	aa	aa	aa	aa	aa	aa	aa	aa
S		Ner	NMB1080	(A) 7	263	aa	aa	aa	aa	aa	aa	aa	aa
S		Putative glycosyl transferase	NMB1255	(G) c	1011	aa	pa	pp	aa	aa	aa	aa	aa
K		Type III restriction system methylase	NMB1261	(CCCAA) 16	2084	aa	pp	pp	pa	aa	aa	pa	aa
S		Mod	NMB1375	(GCCA) 20	2110	aa	aa	aa	aa	aa	aa	aa	aa
S		NifS	NMB1379	(C) 8	1215	pa	pa	pa	pa	pa	pa	pa	pa
S		RTX-type toxin	NMB1407	(ATAACAAA) 4	1130	aa	aa	aa	aa	aa	aa	aa	aa
**K**	***porA***	**Outer membrane protein**	**NMB1429**	**(G) 11**	**1179**	**p?**	**aa**	**aa**	**p?**	**p?**	**p?**	**p?**	**aa**
S		Ppx	NMB1467	(C) c	1509	pp	pa	pa	pa	pa	pa	pa	pp
S		Hypothetical protein	NMB1507	(CAAG) 11	360	aa	pp	pp	aa	aa	aa	aa	aa
S	*lbpA*	Lactoferrin-binding protein A	NMB1540	(G) 8	2832	pa	aa	pa	pa	aa	aa	aa	aa
S		Phage associated	NMB1543	(C) 8 (N) 10 (G)	1215	aa	aa	pa	pa	aa	aa	aa	aa

				7									
S		Phage associated	NMB1634	(C) 8 (N) 9 (G) 7	1209	aa	aa	pa	pa	aa	aa	aa	aa
K	*hmbR*	Hemoglobin receptor	NMB1668	(G) 10	2376	aa	pa	aa	aa	pa	pa	aa	pa
S		FixP	NMB1723	(AT) 5	1098	pa	pp	pp	pa	pp	pp	pp	pa
S		Phage associated	NMB1741	(C) 6 (N) 10 (G)7	1343	aa	aa	pp	pa	aa	aa	aa	aa
S		CvaA	NMB1783	(C) 5	1225	aa	aa	aa	aa	aa	aa	aa	aa
**S**		**Hypothetical protein**	**NMB1786**	**(A)**	**1155**	**p?**	**aa**	**aa**	**aa**	**aa**	**aa**	**aa**	**aa**
K	*pglH*	Pilin glycosylation protein	NMB1819	(C) 10	156	aa	pa	aa	aa	aa	pa	pa	aa
S	*wbpC /pglI*	Putative lipopolysaccharide biosynthesis or pilin glycosylation protein	NMB1836	(G) 13	1872	aa	pa	pp	aa	aa	aa	pa	aa
K	*lgtA*	Acto-*N*-neotetraose biosynthesis glycosyl transferase	NMB1929	(G) 11	1050	pa	pp	pp	aa	aa	aa	aa	pa
S		Hypothetical protein	NMB1931	(G) 7	348	pa	pa	aa	aa	aa	aa	aa	aa
S	*aspA*	Putative serotype-1-specific antigen	NMB1969	(C) 10	3249	aa	aa	aa	aa	aa	aa	aa	aa
**K**	***frpB/ fetA***	**Iron-regulated outer membrane protein**	**NMB1988**	**(C) 11**	**2145**	**p?**	**p?**	**p?**	**p?**	**p?**	**p?**	**p?**	**p?**
**K**	***nadA***	**Putative adhesin / invasin**	**NMB1994**	**(TAAA) 9**	**1095**	**p?**	**aa**	**aa**	**aa**	**aa**	**p?**	**p?**	**aa**
S		Map	NMB2093	(A) 7	780	pa	pp	pp	pa	pa	pa	pa	pa
S		MafA-3	NMB2104	(G) c	964	pa	pa	pa	pa	aa	aa	aa	aa
K	*pilC2*	Pilin biogenesis protein	NMC0033	(G) 11 & (G) c	3147	aa	aa	pa	aa	aa	aa	aa	aa
S	*mtfB*	ABC transporter ATP-binding component	NMC0086	(G) 7	2087	aa	aa	aa	aa	aa	aa	aa	aa
K	*pilC1*	Pilin biogenesis protein	NMC0371	(G) c	3117	pa	pa	aa	pa	aa	aa	aa	aa
S	*pglB*2	Pilin glycosylation protein	NMC0399	(A) c	1579	pa	pa	pa	pa	pp	pp	pp	pa
K	*pglG*	Pilin glycosylation protein	NMC0401	(C) 7	1084	pa	aa	pa	pa	pa	pa	pa	pa
K	*pglE*	Pilin glycosylation protein	NMC0568	(CAAACAA) 34 or (CAAACAC) 26	1180	aa	pp	pp	aa	aa	aa	aa	aa

S		Hypothetical protein	NMC0804	(G) c	249	aa	aa	aa	aa	aa	aa	aa	aa
K		Putative restriction system methylase	NMC1310	(GCCA) 20	2235	aa	aa	pp	aa	aa	aa	aa	aa
S	*virG*	AIDA-related protein	NMC1454	(AAGC) 5	1945	aa	aa	aa	aa	aa	aa	aa	aa
K	*hpuA*	Hemoglobin-haptoglobin utilization protein	NMC1946	(G) 10	1026	pa	pp	pp	pa	aa	aa	pa	aa
K	*lgtG*	Lipopolysaccharide glycosyl transferase	NMC2011	(C) 11	1059	aa	aa	pa	aa	aa	aa	aa	pa
S		Putative acetyltransferase	NMC2013	(G) 12	572	aa	aa	aa	aa	aa	aa	aa	aa
K		Hypothetical protein	XNG0503a	(C) 6 (AC) 9	318	aa	aa	pa	aa	aa	aa	aa	aa
K		Hypothetical protein	XNG1733	(G) 8	1062	aa	aa	aa	aa	aa	aa	aa	aa
K		Hypothetical protein	XNG1834	(C) 8	261	aa	pa	pp	aa	aa	aa	aa	aa
K	*lgtC*	Lipopolysaccharide biosynthesis protein	XNG2047	(G) 14	924	aa	aa	aa	aa	aa	aa	aa	aa
K	*lgtD*	Lipopolysaccharide biosynthesis protein	XNG2048	(G) 14	1014	aa	aa	aa	aa	aa	aa	aa	aa
