# Supplementary material for: Genome Sequencing Reveals Widespread Virulence Gene Exchange among Human Neisseria Species
Source: PLoS One. 2010 Jul 28;5(7):e11835. doi: 10.1371/journal.pone.0011835 (PMC2911385; doi:10.1371/journal.pone.0011835)
Supplement: Table S4 — Genes known or hypothesized to be involved in Neisseria virulence. The 177 targets were identified by searching the literature for genes previously identified as having a role in Neisseria virulence. Genes in bold type are present in all 19 Neisseria genomes used in the study. (0.13 MB PDF) [file pone.0011835.s007.pdf]

Table S4. Genes known or hypothesized to be involved in *Neisseria* virulence. The 177 targets were identified by searching the literature for genes previously identified as having a role in *Neisseria* virulence. Genes in bold type are present in all 19 *Neisseria* genomes used in the study.

**Gene Names**				
basR	*iga2*	NGO1755 (Maf-related protein)	**NMB1946 (outer membrane protein)**	***pilO***
basS	***kat***	*nlpD*	**NMB1989 (iron transport-related protein)**	***pilP***
bcp	***kdsA***	NMB0051 (twitching motility protein)	**NMB1990 (iron transport-related protein)**	*pilQ*
bfrA	***kdsB***	NMB0065 (hypothetical protein)	**NMB1991 (iron transport-related protein)**	*pilS*
bfrB	***kdtA***	NMB0293 (TonB-dependent receptor)	**NMB2127 (protease)**	***pilT1***
ctrA	*lbpA*	NMB0364 (FrpC-related protein)	NMB2132 (transferrin-binding protein)	***pilT2***
ctrB	*lbpB*	NMB0365 (FrpC-related protein)	NMW0547 (TonB-dependent receptor)	***pilU***
ctrC	***lgt***	NMB0374 (MafB-related protein)	***norB***	***pilV***
ctrD	*lgtA*	NMB0493 (hemagglutinin/hemolysin-related protein)	***norM* (NMB0812)**	*pilV2*
dca/pptA	*lgtB*	NMB0496 (hemolysin activator-related protein)	*nspA*	***pilW***
dsbA-1	*lgtB2*	NMB0497 (hemagglutinin/hemolysin-related protein)	***nth***	***pilX***
dsbA-2	*lgtE*	NMB0584 (FrpC-related protein)	*oafA*	*porA*
dsbA-3	*lgtF*	NMB0585 (FrpA-related protein)	***omp85***	*porB*
envA	*lipA*	**NMB0586 (adhesin)**	***ompH***	*prc*
farA	*lipB*	NMB0653 (MafB-related protein)	*opa* (NMB0926)	***rfaC***
farB	***lpxA***	NMB0718 (HemH-related protein)	*opa* (NMB1465)	***rfaD***
farR	***lpxB***	NMB0757 (HemH-related protein)	*opa* (NMB1636)	***rfaE***
fbpA	***lpxC***	**NMB0888 (PilW-related protein)**	*opa* (NMB0442)	***rfaF***
fbpB	***lpxD***	NMB1210 (putative toxin-activating protein)	*opcA*	*rfaK*
fbpC	*lst*	NMB1214 (hemagglutinin/hemolysin-related protein)	*opcB*	*rmpM*
fetA	*macA*	NMB1403 (FrpA-related protein)	***penA***	*sacA*

fetB	*mafA-1*	NMB1405 (FrpA-related protein)	*pglA*	*sacB*
fetB2	*mafA-2*	NMB1409 (FrpA-related protein)	*pglB*	*sacC*
fimT	***msbB***	NMB1412 (FrpC-related protein)	***pglC***	***sodB***
fur	*mtrA*	NMB1414 (FrpC-related protein)	*pglD*	*sodC*
galE	***mtrC***	NMB1415 (FrpC-related protein)	***pgm***	*tbpA*
gcp	***mtrD***	**NMB1428 (putative aminopeptidase)**	***phoP***	*tbpB*
glmU	***mtrE***	NMB1646 (putative hemolysin)	***phoQ***	*tonB*
gpxA	*mtrF*	NMB1738 (secretion protein)	*pilC1*	*tspA*
hap	***mtrR***	NMB1753 (VapD-related protein)	*pilC2*	*tspB*
hmbR	***mutL***	NMB1763 (putative toxin-activating protein)	***pilD***	***vacJ***
hpuA	***mutS***	NMB1768 (hemagglutinin/hemolysin-related protein)	***pilE***	*vapA*
hpuB	***nadA***	NMB1780 (hemolysin activator-related protein)	***pilF***	*virG*
hsf	*narE*	**NMB1829 (TonB-dependent receptor)**	***pilG***	
htrB	*natC*	**NMB1870 (GNA1870)**	***pilM***	
iga1	NGO1177 (pilin-related protein)	NMB1882 (TonB-dependent receptor)	***pilN***	
