# Supplementary material for: Genome Sequencing Reveals Widespread Virulence Gene Exchange among Human Neisseria Species
Source: PLoS One. 2010 Jul 28;5(7):e11835. doi: 10.1371/journal.pone.0011835 (PMC2911385; doi:10.1371/journal.pone.0011835)
Supplement: Table S5 — Copy number of pilE, pilS and opa genes in Neisseria species. See supplemental references (Text S1) for additional information on N. meningitidis MC58 [3], [20] and N. gonorrhoeae FA1090 [20], [21]. (0.08 MB PDF) [file pone.0011835.s008.pdf]

Table S5. Copy number of *pilE*, *pilS* and *opa* genes in *Neisseria* species. See supplemental references (Text S1) for additional information on *N. meningitidis* MC58 [3,20] and *N. gonorrhoeae* FA1090 [20,21].

**Genome**	***pilE***	***pilS***	***Opa***
N. elongata	1	5*	0
N. sicca	1	5	0
N. mucosa	1	5	0
N. subflava	2	2	0
N. flavescens	1	4	2
N. cinerea	2	3	0
N. polysaccharea	1	3	1
N. lactamica 23970	1	3	3
N. meningitidis MC58	1	9	4
N. gonorrhoeae FA1090	1	19	11

*The *pilS* copy upstream of *pilE* in *N. elongata* contains a complete open reading frame, but appears to be missing the promoter region.
