# Supplementary material for: Genome Sequencing Reveals Widespread Virulence Gene Exchange among Human Neisseria Species
Source: PLoS One. 2010 Jul 28;5(7):e11835. doi: 10.1371/journal.pone.0011835 (PMC2911385; doi:10.1371/journal.pone.0011835)
Supplement: Table S6 — Recombination of virulence genes among Neisseria species. The RDPv3.18 program was used to test for recombination in 69 virulence genes that are shared by all Neisseria species. (0.13 MB PDF) [file pone.0011835.s009.pdf]

Table S6. Recombination of virulence genes among *Neisseria* species. The RDPv3.18 program was used to test for recombination in 69 virulence genes that are shared by all *Neisseria* species.

Gene	Evidence of Recombination	Recombination in Commensals	Major Parent	Minor Parent	Daughter	*p*-value
NMB1946	Yes	Yes	*N. sicca*	*N. flavescens*	*N. elongata*	$2.76x10^{-6}$
bfrB	Yes	Yes	*N. meningitidis* alpha275	*N. cinerea*	*N. meningitidis* alpha275	$1.4x10^{-4}$
norB	Yes	Yes	*N. flavescens*	*N. polysaccharea*	*N. mucosa*	$5.97x10^{-6}$
pglC	Yes	Yes	*N. meningitidis* alpha275	*N. mucosa*	*N. meningitidis* 053442	$3.1x10^{-7}$
NMB1990	Yes	Yes	*N. subflava*	*N. meningitidis* alpha275	*N. flavescens*	$4.36x10^{-3}$
NMB1991	Yes	Yes	*N. subflava*	*N. lactamica* 23970	*N. meningitidis* FAM18	$3.88x10^{-9}$
NMB2127	Yes	Yes	*N. gonorrhoeae* FA1090	*N. cinerea*	*N. lactamica* 23970	$8.19x10^{-5}$
lpxC	Yes	Yes	*N. gonorrhoeae* MS11	*N. lactamica* 23970	*N. polysaccharea*	$8.3x10^{-6}$
kat	Yes	Yes	*N. mucosa*	*N. lactamica* 23970	*N. gonorrhoeae* FA1090	$7.2x10^{-5}$
NMB1880 (*fetB2)*	Yes	Yes	*N. mucosa*	*N. polysaccharea*	*N. cinerea*	$1.65x10^{-14}$
dsbA-3	Yes	Yes	*N. meningitidis* Z2491	*N. cinerea*	*N. polysaccharea*	$4.07x10^{-3}$
NMB0594 (*phoP*)	Yes	Yes	*N. gonorrhoeae* FA1090	*N. lactamica* 23970	*N. meningitidis* alpha275	$5.8x10^{-12}$
kdtA	Yes	Yes	*N. cinerea*	*N. lactamica* 020-06	*N. meningitidis* 053442	$1.88x10^{-10}$
mutS	Yes	Yes	*N. meningitidis*	*N. lactamica*	*N. meningitidis*	$7.3x10^{-7}$

			FAM18	23970	MC58	
pilT2	Yes	Yes	*N. lactamica* 23970	*N. meningitidis* MC58	*N. meningitidis* FAM18	$3.6x10^{-4}$
pgm	Yes	Yes	*N. subflava*	*N. meningitidis* 053442	*N. meningitidis* alpha275	$1.6x10^{-5}$
NMB0812	Yes	Yes	*N. sicca*	*N. subflava*	*N. lactamica* 23970	$4.21x10^{-4}$
NMB0890 (*pilX*)	Yes	Yes	*N. cinerea*	*N. meningitidis* FAM18	*N. polysaccharea*	$3.55x10^{-5}$
lgt	Yes	Yes	*N. gonorrhoeae* FA1090	*N. sicca*	*N. meningitidis* Z2491	$1.65x10^{-3}$
kdsA	Yes	Yes	*N. lactamica* 23970	*N. meningitidis* alpha275	*N. meningitidis* MC58	$1.6x10^{-4}$
mutL	Yes	Yes	*N. cinerea*	*N. lactamica* 23970	*N. meningitidis* alpha14	$3.9x10^{-9}$
rfaF	Yes	Yes	*N. lactamica* 23970	*N. meningitidis* MC58	*N. polysaccharea*	$8.6x10^{-7}$
mtrE	Yes	Yes	*N. sicca*	*N. flavescens*	*N. mucosa*	$4.26x10^{-6}$
mtrD	Yes	Yes	*N. lactamica* 020-06	*N. gonorrhoeae* NCCP11945	*N. meningitidis* MC58	$2.0x10^{-8}$
mtrC	Yes	Yes	*N. mucosa*	*N. polysaccharea*	*N. cinerea*	$1.46x10^{-7}$
penA	Yes	Yes	*N. gonorrhoeae* FA1090	*N. lactamica* 23970	*N. polysaccharea*	$5.0x10^{-13}$
pilD	Yes	Yes	*N. meningitidis* alpha14	*N. lactamica* 23970	*N. meningitidis* MC58	$3.85x10^{-6}$
pilF	Yes	Yes	*N. meningitidis* alpha275	*N. lactamica* 23970	*N. polysaccharea*	$2.56x10^{-3}$
farB	Yes	Yes	*N. lactamica* 020-06	*N. polysaccharea*	*N. lactamica* 23970	$5.59x10^{-3}$
lpxD	Yes	Yes	*N. lactamica*	*N. meningitidis*	*N. meningitidis*	$7.7x10^{-3}$

			23970	053442	alpha275	
glmU	Yes	Yes	*N. meningitidis* 053442	*N. sicca*	*N. cinerea*	$7.4x10^{-8}$
rfaC	Yes	Yes	*N. meningitidis* MC58	*N. lactamica* 23970	*N. lactamica* 020-06	$1.96x10^{-7}$
omp85	Yes	Yes	*N. meningitidis* Z2491	*N. cinerea*	*N. meningitidis* alpha14	$7.19x10^{-8}$
pilO	Yes	Yes	*N. polysaccharea*	*N. cinerea*	*N. meningitidis* 053442	$3.82x10^{-3}$
NMB1870	Yes	No	*N. meningitidis* alpha14	*N. meningitidis* MC58	*N. gonorrhoeae* FA1090	$1.28x10^{-4}$
NMB0825 (*rfaE*)	Yes	No	*N. meningitidis* MC58	Unknown	*N. meningitidis* Z2491	$9.6x10^{-7}$
NMB1961 (*vacJ*)	Yes	No	*N. gonorrhoeae* FA1090	Unknown	*N. gonorrhoeae* NCCP11945	$7.959x10^{-12}$
nadA	Yes	No	Unknown	*N. polysaccharea*	*N. elongata*	$1.1x10^{-11}$
nth	Yes	No	Unknown	*N. meningitidis* alpha14	*N. meningitidis* 053442	$3.2x10^{-3}$
NMB1801 (*htrB*)	Yes	No	*N. meningitidis* 053442	*N. meningitidis* MC58	*N. meningitidis* alpha275	$4.74x10^{-5}$
kdsB	Yes	No	*N. gonorrhoeae* FA1090	*N. meningitidis* 053442	*N. meningitidis* alpha275	$7.66x10^{-3}$
rfaD	Yes	No	Unknown	*N. meningitidis* alpha14	*N. meningitidis* alpha275	$1.10x10^{-8}$
fimT	Yes	No	*N. meningitidis* MC58	*N. gonorrhoeae* NCCP11945	*N. meningitidis* 053442	$4.14x10^{-4}$
NMB0887 (*pilV*)	Yes	No	*N. meningitidis* 053442	Unknown	*N. gonorrhoeae* NCCP11945	$3.06x10^{-6}$
NMB0888 (*pilW*-related)	Yes	No	*N. meningitidis* MC58	*N. gonorrhoeae* FA1090	*N. meningitidis* 053442	$5.62x10^{-8}$

NMB1989	Yes	No	Unknown	*N. gonorrhoeae* MS11	*N. polysaccharea*	$1.2x10^{-9}$
NMB1418 (*msbB*)	Yes	No	*N. meningitidis* alpha275	*N. meningitidis* MC58	*N. meningitidis* FAM18	$7.58x10^{-6}$
NMB1428	Yes	No	*N. meningitidis* alpha14	*N. meningitidis* 053442	*N. meningitidis* Z2491	$6.47x10^{-6}$
fetA	Yes	No	*N. gonorrhoeae* FA1090	*N. meningitidis* alpha275	*N. gonorrhoeae* MS11	$1.5x10^{-8}$
lpxB	Yes	No	*N. meningitidis* alpha14	*N. meningitidis* MC58	*N. meningitidis* FAM18	$3.39x10^{-4}$
lpxA	Yes	No	*N. meningitidis* Z2491	Unknown	*N. meningitidis* 053442	$8.91x10^{-4}$
NMB0586	Yes	No	*N. meningitidis* Z2491	Unknown	*N. gonorrhoeae* NCCP11945	$5.906x10^{-3}$
gcp	Yes	No	*N. meningitidis* alpha14	*N. meningitidis* alpha275	*N. meningitidis* MC58	$5.07x10^{-3}$
bfrA	No	No	NA	NA	NA	NA
NMB1829	No	No	NA	NA	NA	NA
bcp	No	No	NA	NA	NA	NA
sodB	No	No	NA	NA	NA	NA
NMB0181 (*ompH*)	No	No	NA	NA	NA	NA
NMB0595 (*phoQ*)	No	No	NA	NA	NA	NA
pilW	No	No	NA	NA	NA	NA
mtrR	No	No	NA	NA	NA	NA
pilG	No	No	NA	NA	NA	NA
fur	No	No	NA	NA	NA	NA
NMB1843 (*farR*)	No	No	NA	NA	NA	NA

pilT1	No	No	NA	NA	NA	NA
pilU	No	No	NA	NA	NA	NA
pilP	No	No	NA	NA	NA	NA
pilN	No	No	NA	NA	NA	NA
pilM	No	No	NA	NA	NA	NA
