# Supplementary material for: Genome Sequencing Reveals Widespread Virulence Gene Exchange among Human Neisseria Species
Source: PLoS One. 2010 Jul 28;5(7):e11835. doi: 10.1371/journal.pone.0011835 (PMC2911385; doi:10.1371/journal.pone.0011835)
Supplement: Table S7 — Primers used for PCR amplification and sequencing of potential pseudogenes. (0.07 MB PDF) [file pone.0011835.s010.pdf]

Table S7. Primers used for PCR amplification and sequencing of potential pseudogenes.

Primer	Gene	Species	Sequence
MR13	*hpuA*-F	*N. lactamica*	ATGAAATACAAAGCCCTGTCCTTAC
MR14	*hpuA*-R		AGGTCGATTTCGCCGTTG
MR15	*hmbR*-F	*N. polysaccharea*	ATCGATACCGAGTTGGTTAAGG
MR16	*hmbR*-R		ATAACCTGTGCGCCAGTCTC
MR17	*hpuB*-F	*N. flavescens*	ATGCCGAAAAACAAATCGAC
MR18	*hpuB*-R		TTGGGGTTGGCTTTAAGGTAG
MR19	*pilQ*-F	*N. cinerea*	CGAACTCGGTTCCCTCTATTC
MR20	*pilQ*-R		TACCAGTGCGATGCTGTTTG
MR21	*pilU*-F	*N. flavescens*	GGCAGCTTGAGACCTTCAAC
MR22	*pilU*-R		ACAGATGGCCGGTTTGAG
MR23	*pilN*-F	*N. cinerea*	AATTAAAATTAACCTTCTTCCCTACAG
MR24	*pilN*-R		TCTCAGAAGCCTTTACGATGG
MR25	*pilN*-F	*N. subflava*	CAGAATCAACCTTCTCCCATATC
MR26	*pilN*-R		CCTGCGTATTCACTTGGTTG
MR27	*lipA*-F	*N. subflava*	TTTTGCCGAACAACATAATATCC
MR28	*lipA*-R		ATCAGCTGCCAAAGCTCAAG
